# Relationship Between Subjective Ratings of Answers and Behavioral and Autonomic Nervous Activities During Creative Problem-Solving *via* Online Conversation

**DOI:** 10.3389/fnins.2021.724679

**Published:** 2021-10-04

**Authors:** Takashi Numata, Kiyoshi Kotani, Hiroki Sato

**Affiliations:** ^1^Research & Development Group, Hitachi, Ltd., Kokubunji, Japan; ^2^Research Center for Advanced Science and Technology, The University of Tokyo, Meguro, Japan; ^3^Department of Bioscience and Engineering, Shibaura Institute of Technology, Saitama, Japan

**Keywords:** online communication, creative problem solving, subjective confidence, behavioral activity, autonomic nervous activity

## Abstract

Creative problem solving has been important for the advent of new technologies. In this study, we hypothesized that subjective ratings of answers should be useful for evaluating the answer quality in creative problem solving. To test this hypothesis and extract objective indicators of the subjective ratings of answers, we evaluated the relationship between subjective ratings of task performance and behavioral and autonomic nervous activities during a creative problem-solving task performed *via* online conversation. The task involved an answerer and a supporter, and in the experiment, each pair performed 10 trials. The trials were categorized as highly or lowly rated according to the answerer’s confidence in the answer. The task performance and behavioral and autonomic nervous activities were then compared between these categories of trials. Behavioral activity was evaluated *via* movements and speech activities, while for autonomic nervous activity, sympathetic nervous activity (SNA) was evaluated *via* skin conductance. The task performance was significantly better in the highly rated trials, whereas there were no significant differences in the behavioral activities between the highly and lowly rated trials. Moreover, in the highly rated trials, the skin conductance of the answerer was significantly high, whereas that of the supporter was significantly low. The results support the hypothesis and suggest that contrasting differences in SNA between an answerer and a supporter are indicators of the subjective ratings of answers in creative problem solving.

## Introduction

Although recent technologies have taken advantage of the progress in artificial intelligence, robotics, and big data, the human capacity for creative problem solving remains superior to that of artificial intelligence. Creative problem solving involves solving a problem when the correct answer, appropriate approach, and solution method are unknown (Carmeli et al., 2014; Cybulski et al., 2015). Accordingly, development of this capacity and support for creative problem solving are crucial to the advent of new technologies.

In the process of creative problem solving, not only logical thinking but also ideation and inspiration are important (Carmeli et al., 2014; Cybulski et al., 2015). However, it is quite difficult to evaluate the quality of ideation and inspiration by evaluating logical consistency and/or analyzing data, and thus, it is also difficult to do so for creative problem solving. In practice, people subjectively evaluate the quality of an idea or inspiration, choose an approach and a solution method, and decide on the answer (Carmeli et al., 2014; Cybulski et al., 2015). Although subjective evaluation is another human capacity that is superior to that of artificial intelligence, the relationship between subjective ratings of answers and the quality of the answers has not yet been fully validated. Accordingly, it would be useful to understand this relationship in the context of the creative problem solving.

In addition, to both develop the capacity for and support creative problem solving, learning through communication with teachers has long been the main approach in the field of education (Lumsdaine and Lumsdaine, 1994; Kandemir and Gür, 2009). More generally, conversation is widely used to improve the quality of creative problem solving (Carmeli et al., 2014; Cybulski et al., 2015). In particular, the use of online conversation has increased worldwide because of the COVID-19 pandemic, so it has become more important to improve the quality of creative problem solving done *via* online conversation. However, online conversation often reduces communication quality because of network delays and disturbances and the limited amount of non-verbal information (Mallen et al., 2003), which hinders ideation and inspiration. In this situation, it is more difficult to communicate subjective perceptions and expressing notions such as explanations of subjective ratings is more difficult than in face-to-face communication. Accordingly, to visualize subjective information and facilitate smooth online conversation, it would be useful to extract objective indicators of the subjective ratings of answers in creative problem solving.

Regarding subjective information, behavioral and autonomic nervous activities should be useful for extracting effective indicators. Both activities are known as indicators of various kinds of subjective information such as subjective feelings and emotion (Ekman et al., 1980; Bänziger et al., 2009; Kreibig, 2010). Behavioral activities such as facial expressions, body movements, and speech activities have the advantage of enabling non-contact measurement. Physiological activities such as autonomic nervous activity have the advantage of providing deeper information about people, making it possible to clarify the physiological mechanisms of subjective feelings and emotions. Hence, we assumed that such behavioral and autonomic nervous activities can contribute to the identification of objective indictors for the subjective ratings of answers in creative problem solving. These indicators would then enable us to develop technologies for visualizing the subjective ratings of answers and improving the quality of answers.

With the goal of enhancing the capacity for creative problem solving *via* online conversation, our study had two objectives. Objective (1) was to confirm the relationship between the subjective ratings of answers and the quality of answers. For objective (1), estimation tasks involving Fermi questions (i.e., Fermi estimation tasks) are useful as a source of creative problem-solving tasks. A Fermi question entails the problem of estimating a complex quantity quickly without information on the actual quantity (Efthimiou and Llewellyn, 2007; Anderson and Sherman, 2010). Such estimation requires setting up a reasonable hypothesis and using logic (Efthimiou and Llewellyn, 2007; Anderson and Sherman, 2010), as with creative problem solving. In addition, the Fermi estimation tasks have the advantage of enabling performance evaluation by calculating a task score based on an actual quantity. Accordingly, these tasks enable objective evaluation of performance in creative problem solving, and, in turn, of the relationship between the task performance and the subjective ratings of answers. Next, objective (2) was to extract objective indicators of the subjective ratings of answers in creative problem solving done *via* online conversation. For objective (2), behavioral and autonomic nervous activities were measured during the Fermi estimation tasks performed *via* online conversation, and the relationships between the subjective ratings of answers and the behavioral and autonomic nervous activities were evaluated.

## Materials and Methods

### Experimental Procedures

Ten healthy Japanese men with a mean age of 23.9 ± 1.9 years participated in the experiment. The Fermi estimation task in the experiment involved a pair consisting of an answerer and a supporter. The role of the answerer was to decide an answer to a question, and that of the supporter was to give the answerer advice to improve the answer *via* online conversation. In detail, the answerer was given a question of Fermi estimation, given a chance to seek advice from the supporter *via* online conversation, and asked to decide an answer to the question after the conversation. The supporter was only asked to give advice, with no information besides what the answerer told him. Each participant was asked to play the role of the answerer and the supporter once each, giving a total of 10 pairs. All the participants knew each other as friends before the experiment.

The task consisted of two periods: an individual answer period and a pair improvement period. In the individual answer period, the answerer was told to estimate a quantity (e.g., the number of cars in Japan) alone. Each period included five estimation questions (trials). The individual answer period was followed by the pair improvement period, in which the answerer could improve his answers *via* online conversation with the supporter. The durations of the individual answer period and the pair improvement period were 60 and 120 s, respectively. The answerer was told to write an answer on a piece of paper within 10 s after each period. During the task, pens, a calculation sheet, and a calculator were provided to the answerer. He could use them anytime in the individual answer period and in the last 30 s of the pair improvement period in order to encourage conversation. In comparison, the supporter had no tools or information about the questions. Therefore, the answerer was supposed to share the presented question and related information. We prepared 70 Fermi questions, and the questions in each experiment were randomly chosen.

**FIGURE 1:**
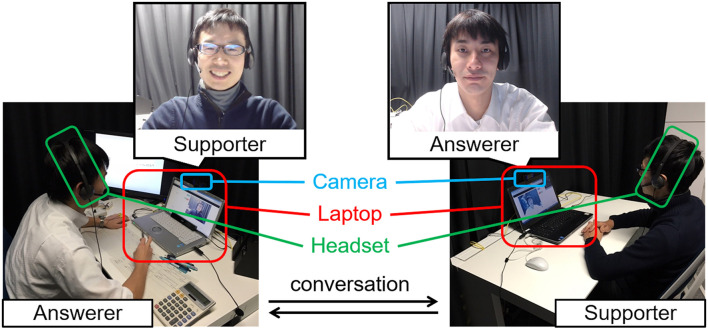
Communication system used during the pair improvement period in the creative problem-solving task.

To enable online conversation between the answerer and supporter during the pair improvement period, we developed a communication system using two laptops equipped with web cameras (c920, Logicool) and headsets (h340, Logicool) as shown in Figure 1. In this system, the laptops were placed in front of the answerer and the supporter, who then used the equipment to convey their facial expressions, body movements, and voices to each other. The application for real-time communication *via* the laptops was developed using WebRTC (Web Real-Time Communication).

### Measurements

The measurements included both subjective ratings and measurements of the task performance and behavioral and autonomic nervous activities. Subjective ratings were obtained for both objectives (1) and (2), the task performance was measured for objective (1), and the behavioral and autonomic nervous activities were measured for objective (2). The protocol was approved by the Ethics Committee of the Research Center for Advanced Science and Technology at The University of Tokyo. The data were obtained in accordance with the standards of the internal review board of the Research & Development Group, Hitachi, Ltd., following receipt of the participants’ informed consent.

For the subjective ratings, the confidence in an answer was evaluated for each trial. The confidence was rated by using a visual analog scale (VAS) (Åkerstedt and Gillberg, 1990; Torrance et al., 2001): each answerer was told to express his confidence in an answer by drawing a vertical line to meet a horizontal lines whose left and right ends were set to 0 and 100, respectively. For this confidence evaluation, the description at the left end was “does not feel confident at all” and that at the right end was “feels very confident” (in the participants’ first language). These subjective ratings were taken immediately after an answer was written down in each trial.

The closeness of an answer to the actual data value was calculated as the task score. Specifically, the equation for the task score was


(1) $$\text{score}=\log{\text{x}}_{\mathrm{actual}}-\log{\text{x}}_{\mathrm{answer}},$$

where ***x*_actual_** was the actual value, and ***x*_answer_** was the answer in the pair improvement period. Thus, the task score was calculated by subtracting answers from the actual data on a logarithmic scale.

The evaluated behavioral activities were movements of the head and body in the videos captured by the cameras and the speech activities captured from the participants’ voices. These activities are often shared smoothly *via* face-to-face conversation but are difficult to share *via* online conversation, and they are known to be affected by a person’s mental condition (Ekman et al., 1980; Bänziger et al., 2009). Therefore, we expected the experiment to reveal differences in behavioral activity depending on the subjective ratings of answers. The head and body movements and voices data were measured *via* the online conversation system during the pair improvement period. The sampling rate of the video measurement was 30 Hz, and that of the voice measurement was 44,100 Hz.

**FIGURE 2:**
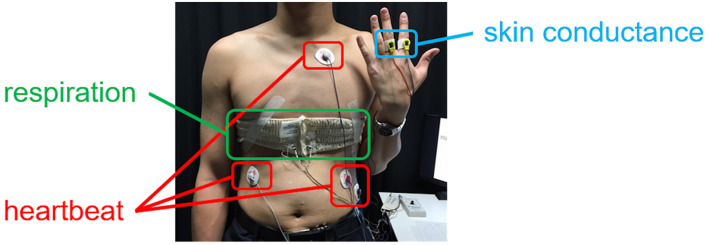
Measurements of the heartbeat, respiration, and perspiration. The heartbeat was obtained from an ECG signal, and the perspiration was evaluated *via* the skin conductance.

The evaluated autonomic nervous activities were those of the autonomic nervous system (ANS). The ANS has the important role of maintaining homeostasis in humans. As noted above, it is known to be affected by a person’s mental condition (Kotani et al., 2007b; Kreibig, 2010), and accordingly, the experiment was expected to reveal differences in ANS activity due to the subjective ratings. To evaluate ANS activity, the heartbeat, respiration, and perspiration were measured during the pair improvement period. As shown in Figure 2, the heartbeat was obtained by measuring an electrocardiogram (ECG: Polymate II and AP-C311-015, Miyuki Giken), respiration was measured with an inductive plethysmograph (Portable Respitrace, A.M.I), and perspiration was measured in terms of the skin conductance (EDA Unit AP-U030, Miyuki Giken) between the index and middle fingers of the non-dominant hand. The sampling rates of these measurement devices were all 1,000 Hz.

### Data Analysis

In this study, we defined highly and lowly rated conditions based on of the subjective confidence of answers in creative problem solving, collected highly and lowly rated trial data, and compared the improvements in the task performance and the changes in behavioral and autonomic nervous activities between the highly and lowly rated trials. By differentiating highly and lowly rated trials for the same participants, we could suppress the influence of interindividual variability (in particular, of the autonomic nervous activities induced by the individual differences in physiological characteristics).

The subjective confidence ratings in the pair improvement period were used to divide the trials into highly and lowly rated ones. The confidence threshold was individually set to the average value for each participant; thus, a highly rated trial was defined as one with a higher confidence than the threshold, while a lowly rated trial was defined as one with a lower confidence than the threshold.

Regarding objective (1), to evaluate the relationship between the subjective ratings and task performance, the task scores in the pair improvement period were compared in terms of the subjective confidence in the answers.

As for objective (2), the behavioral and ANS activities were analyzed. The behavioral activity consisted of head and body movements and speech activities. The amount of head and body movements were evaluated together by using motion energy analysis (Grammer et al., 1999; Ramseyer and Tschacher, 2011, 2014). Motion energy was defined in terms of the frame-by-frame differences in gray-scale pixels between two continuous images, which enabled us to quantify head and body movements in videos. The total amount of head and body movements was derived from each trial by averaging the motion energy in the trials. In addition, the speech duration, speech energy, speech number, and short speech number were evaluated as indicators of speech activity. First, speech activity was detected as a combination of the short-term energy (STE) and zero crossing rate (ZCR) (Lokhande et al., 2012; Jalil et al., 2013; Muzahid et al., 2016). The STE was calculated as:


$$\text{STE}\,\left(m\right)=\frac{1}{\text{L}}\,\sum_{n=m-L+1}^{M}\text{s}\,{\left(n\right)}^{2},$$

and the ZCR was calculated as:


$$\text{ZCR}\,\left(m\right)=\frac{1}{2\,\text{L}}\,\sum_{n=m-L+1}^{M}|\text{sgn}\,\left(\text{s}\,\left(n\right)\right)-\text{sgn}\,\left(\text{s}\,\left(\text{n}-1\right)\right)$$

$$\text{sgn}\,\left(\text{s}\,\left(n\right)\right)=\left\{\begin{array}{c} +1.\text{s}\,\left(n\right)\geq 0\\ --1,\text{s}\left(\text{n}\right)<0 \end{array}\right\},$$

where *L* was the length of a piece of voice time series data. When both the STE and ZCR were larger than their corresponding threshold values, speech activity was detected at that time. After detecting speech activity, the speech duration, speech energy, speech number, and short speech number were calculated. The speech duration was calculated as the sum of the speech activity durations. The speech energy was calculated by averaging the STEs of the speech activity. The speech number was obtained by counting the number of discontinuous speech activities. Here, discontinuous speech activity was defined as speech activity that was separated by at least 1,000 ms from other speech activity. In other words, when a speech activity was detected within 1,000 ms of a previous speech activity, these activities were regarded as a continuous speech activity. Finally, the short speech number was obtained by counting the number of speech activities for which the duration of the activity was within 2,000 ms. Each of these indicators was derived for each pair improvement period.

The ANS activity consisted of sympathetic nervous activity (SNA) and parasympathetic nervous activity (PNA). The SNA and PNA were determined from the heartbeat, respiration, and perspiration during the pair improvement period. Because measurement error prevented collection of the physiological data for one participant, the SNA and PNA of nine participants were analyzed. The SNA was evaluated *via* the skin conductance, which was obtained from the amount of perspiration; that is, the median values of the skin conductance in the pair improvement period were calculated as an indicator of SNA. The PNA was evaluated *via* respiratory sinus arrhythmia (RSA), which is known as an aspect of heart rate variability that is evoked by respiration; accordingly, the amplitude of RSA has been used as an indicator of PNA (Kotani et al., 2007b; Numata et al., 2015). To extract the RSA amplitude with high accuracy, we applied respiratory-phase domain analysis (Kotani et al., 2007b; Numata et al., 2015). This analysis consisted of three steps: finding the beginnings of inspiration and expiration (i.e., the two respiratory phases) for each respiration by using the Hilbert transform; identifying heartbeat intervals at the beginnings of inspiration and expiration by using the derivative in the cubic spline interpolation method; and, in reference to their timings on the basis of the respiratory phases, and calculating the difference in heartbeat intervals between the beginning of inspiration and expiration as the RSA amplitude. In the last step, the median RSA amplitude for each pair improvement period was calculated as the PNA indicator. The signal processing of the respiratory-phase domain analysis was based on the signal processing approaches of previous studies (Kotani et al., 2007b; Numata et al., 2015). For both the signal processing of behavioral and autonomic nervous activities, the signal processing was performed by using MATLAB R2017b (MathWorks, United States).

The scores in the highly and lowly rated trials were compared by statistical analysis with a paired *t*-test. The behavioral activity was evaluated by a two-way ANOVA with simple effects tests of two factors to investigate the effects of the role (answerer or supporter) and the subjective rating (highly or lowly rated trials). Because ANS activity varies significantly depending on the physiological characteristics of individuals, it was quite difficult to compare between the answerer and supporter with the sample size in this study. Accordingly, the ANS activity was also compared between the highly and lowly rated trials with a paired *t*-test. The threshold of significance for the *p*-value was 0.05. The statistical analysis was performed by using HAD (Shimizu, 2016).

## Results

**FIGURE 3:**
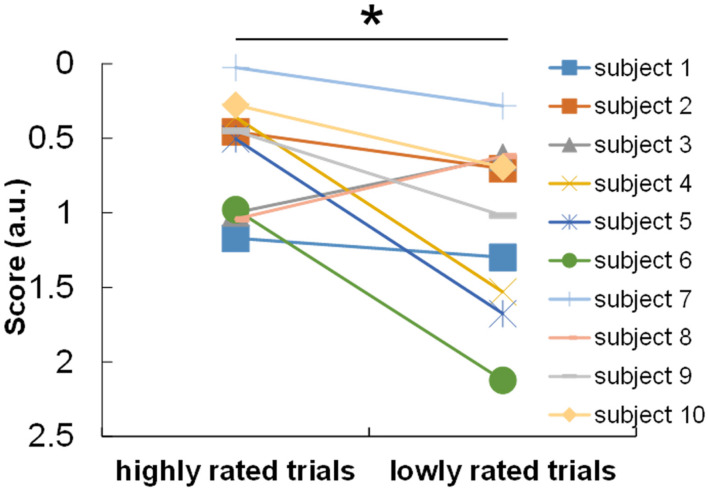
Comparison of task scores between highly and lowly rated trials in terms of subjective confidence in answers. *p < 0.05.

For objective (1), the relationship between the subjective ratings of answers and the task score was evaluated. Comparison of the task scores between the subjectively rated trials showed that the score of the highly rated trials were significantly higher than those of the lowly rated trials [*t*(9) = 2.302, *p* = 0.047] as shown in Figure 3.

**FIGURE 4:**
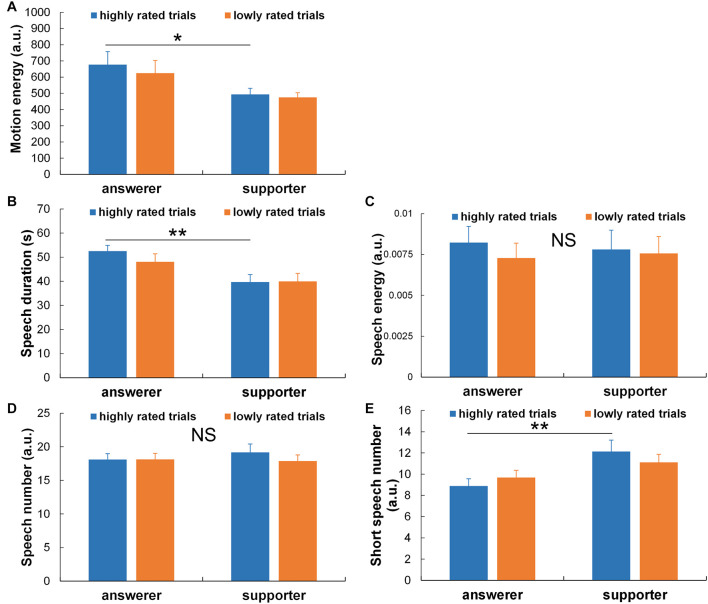
Comparisons of behavioral activities between highly and lowly rated trials in terms of the subjective confidence in answers and between the answerer and supporter roles: **(A)** motion energy, **(B)** speech duration, **(C)** speech energy, **(D)** speech number, and **(E)** short speech number. **p < 0.01; *p < 0.05. NS, not significant.

For objective (2), the relationships between the subjective ratings of answers and the indicators of behavioral and autonomic nervous activities were evaluated. Regarding the behavioral activity, the two-way ANOVA revealed no significant interactions between the role and the subjective ratings in terms of the motion energy, speech duration, speech energy, speech number, or short speech number [respectively, *F*(1,18) = 0.841, *p* = 0.371, η^2^ = 0.045; *F*(1,18) = 1.968, *p* = 0.178, η^2^ = 0.099, *F*(1,18) = 0.809, *p* = 0.380, η^2^ = 0.318; *F*(1,18) = 1.390, *p* = 0.254, η^2^ = 0.072; *F*(1,18) = 2.050, *p* = 0.169, η^2^ = 0.102]. The simple effects test results revealed significant differences only in the highly rated trials. Specifically, as shown in Figure 4, in only the highly rated trials, the motion energy of the answerer was significantly larger than that of the supporter [*t*(36) = 2.132, *p* = 0.040]; the speech duration of the answerer was significantly longer than that of the supporter [*t*(36) = 2.981, *p* = 0.005]; and the short speech number of the answerer was significantly smaller than that of the supporter [*t*(36) = 2.823, *p* = 0.008].

**FIGURE 5:**
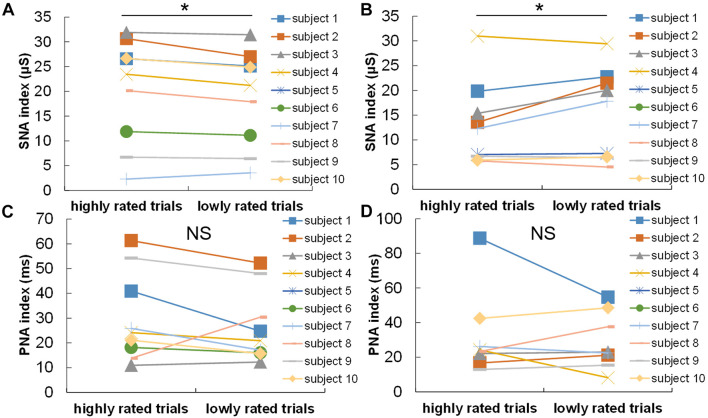
Comparisons of autonomic nervous activities between highly lowly rated trials in terms of the subjective confidence in answers: comparison of **(A)** the answerer’s and **(B)** the supporter’s skin conductance between highly and lowly rated trials; and comparison of **(C)** the answerer’s and **(D)** the supporter’s RSA amplitudes of between highly and lowly rated trials. *p < 0.05. NS, not significant.

Finally, regarding the autonomic nervous activity, the skin conductance of the answerer was significantly higher in highly rated trials than in lowly rated ones [*t*(8) = 2.744, *p* = 0.025]. In contrast, the skin conductance of the supporter was significantly lower in highly rated trials than in lowly rated ones. As shown in Figure 5, the RSA amplitude did not show any significant differences between highly and lowly rated trials (Figure 5) [*t*(8) = 1.219, *p* = 0.258].

## Discussion

In this study, we set two objectives: (1) to confirm the relationship between the subjective ratings and the quality of the answers, and (2) to extract objective indicators of the subjective ratings of answers, obtained from behavioral and autonomic nervous activities, for creative problem solving *via* online conversation. Regarding objective (1), the results showed that the task scores of highly rated trials were better than those of lowly rated trials. In the experiment, neither the correct answers nor the task scores were shown to the answerer and supporter. Therefore, the answerers could not obtain certain information in relation to the answers, and they had to subjectively evaluate their answers completely on their own. As a result, we confirmed that there is a relationship between the task score and the subjective rating of an answer, and this subjective rating should be a useful indicator of the quality of an answer.

As for objective (2), the autonomic nervous activity results showed that the answerer’s skin conductance was high and that of the supporter was low in highly rated trials, as compared with lowly rated trials. Autonomic nervous activity is known to be affected by both physical activity and mental state (Kotani et al., 2007a,b; Numata et al., 2013, 2015; Okawa et al., 2019; Ziemssen and Siepmann, 2019; Park et al., 2020). Because the behavioral results showed no significant differences in the comparison between highly trials and lowly rated trials, the physical activities of the answerer and supporter were not supposed to differ depending on the subjective ratings of answers. Rather, the contrasting differences between the sympathetic activities of the answerer and supporter should have been induced by their mental states. Thus, we suggest that these changes constitute an objective indicator of the subjective ratings of answers in creative problem solving.

This suggested objective indicator, based on contrasting differences between the sympathetic nervous activities of the answerer and supporter, should enable us to develop technologies for evaluating and visualizing the subjective ratings of answers. For example, by monitoring such ratings, it would be possible to develop an automatic support system for an online business meeting that requires creative problem solving such as formulating a new business plan or exploring a new product. When contrasting differences between the sympathetic activities of a determiner and an advisor are detected, the support system would automatically infer the current condition by visualizing the estimated subjective ratings of answers from those activities. The system could then provide advice on the meeting to smooth online conversation and improve the meeting’s effectiveness. Such applications could also be useful in other fields such as education and advertising. That is, evaluation and visualization of contrasting differences between the sympathetic nervous activities of an answerer and a supporter could potentially enable technologies for evaluating and visualizing the subjective ratings of answers, smoothing online conversation, and improving the quality of creative problem solving.

The behavioral activities of the answerer and supporter did not show significant differences between highly and lowly rated trials; however, in only the highly rated trials, there were significant results indicating that the motion energy of the answerer was larger, the speech duration of the answerer was longer, and the short speech number of the answerer was smaller, as compared to the supporter. These results imply that in the highly rated trials the answerer was the main speaker and the supporter was an active listener. As a result, the ratios of the motion energy, speech duration and/or short speech number between an answerer and a supporter are likely to be supplementary indicators of the subjective ratings of answers.

From the viewpoint of the physiological relationship between the subjective ratings of answers and autonomic nervous activity, we emphasize that the PNA did not show any significant differences. In most situations, it is known that sympathetic and PNA have opposite actions. Nevertheless, in this study, six of the nine answerers showed higher sympathetic and parasympathetic nervous activities in highly rated trials than in lowly rated trials. In contrast, five of the nine supporters showed higher sympathetic and parasympathetic nervous activities in lowly rated trials than in highly rated trials. Such a short-term coactivation of sympathetic and PNA in cognitive tasks is known to be induced by a flow state (Jackson and Marsh, 1996; Knierim et al., 2018). A flow state is defined as the mental state of being highly focused on and deeply immersed in something, and it is known to lead to better performance on cognitive tasks (Jackson and Marsh, 1996; Knierim et al., 2018). Accordingly, we speculate that the answerer’s mental state was close to a flow state when he felt subjectively confident in his answer, whereas the supporter’s mental state was close to a flow state when the answerer did not feel subjectively confident in his answer.

This study has three main limitations. First, the type of creative problem-solving task was limited to a Fermi estimation task. Although this task has an advantage in that the task performance can be evaluated with actual data, the creativity in the task was mainly focused on the approach process. Second, the situation was limited to conversation between a pair consisting of an answerer and a supporter. In the process of ideation during creative problem solving, multi-person (i.e., more than two people) conversation is useful for improving the quality of creative problem solving (Carmeli et al., 2014; Cybulski et al., 2015). In addition, there are situations in which speakers’ roles are not clearly defined and consensus building is necessary. Third, the indicators of behavioral and physiological activities were limited to indicators that are relatively easy to measure. There are other behavioral activities such as facial expressions measured by electromyogram, and there are other physiological activities such as brain activity measured by electroencephalogram (EEG), near-infrared spectroscopy (NIRS), and/or magnetic resonance imaging (MRI). There is EEG equipment that allows significant head movement during conversation without seriously degrading the signal quality (Hemalatha et al., 2018); however, measurements of these indicators have the disadvantages of reducing the quality of conversation by hiding facial expressions and disrupting natural head movements because of the perceived restriction by attached measurement devices. In this study we prioritized measurement and evaluation of natural head movements during conversation, but these indicators could help us to understand the deeper physiological mechanisms underlying the subjective confidence of answers in creative problem solving. To validate the relationship between the subjective ratings of answers and the quality of the answers, as well as the versatility of the objective indicators proposed in this study, it would be effective to evaluate them through various creative problem-solving tasks with a variety of conversation situations and measurement indicators.

## Conclusion

In this study, we evaluated the relationship between subjective ratings of task performance and behavioral and autonomic nervous activities during a creative problem-solving task performed *via* online conversation. As a result, we confirmed that there is a relationship between subjective ratings of answers and the quality of answers; we also extracted contrasting differences in SNA between an answerer and a supporter as an objective indicator of the subjective ratings of answers. The results suggest that evaluation and visualization of the sympathetic nervous activities of an answerer and a supporter during creative problem solving *via* online conversation would be useful for visualizing subjective information, which could facilitate smooth online conversation and improve the quality of creative problem solving.

## Data Availability Statement

The raw data supporting the conclusions of this article will be made available by the authors, without undue reservation.

## Ethics Statement

The studies involving human participants were reviewed and approved by the Research Center for Advanced Science and Technology, The University of Tokyo. The patients/participants provided their written informed consent to participate in this study. Written informed consent was obtained from the individual(s) for the publication of any potentially identifiable images or data included in this article.

## Author Contributions

TN designed and conducted the experiments, analyzed and interpreted the results, and drafted the manuscript. KK and HS designed the experiment, interpreted the results, and revised the manuscript. All authors contributed to the article and approved the submitted version.

## Conflict of Interest

TN is employed by Hitachi, Ltd. The remaining authors declare that the research was conducted in the absence of any commercial or financial relationships that could be construed as a potential conflict of interest.

## Publisher’s Note

All claims expressed in this article are solely those of the authors and do not necessarily represent those of their affiliated organizations, or those of the publisher, the editors and the reviewers. Any product that may be evaluated in this article, or claim that may be made by its manufacturer, is not guaranteed or endorsed by the publisher.
